# Supplementary material for: A bacterial immunomodulatory protein with lipocalin-like domains facilitates host–bacteria mutualism in larval zebrafish
Source: eLife. 2018 Nov 6;7:e37172. doi: 10.7554/eLife.37172 (PMC6219842; doi:10.7554/eLife.37172)
Supplement: Supplementary file 1. — Mass-spectrophotometry was performed on the CFS from ∆T2C and ∆T2 determined which proteins were enriched in the ΔT2C compared to the ΔT2 strain. This table lists the top 22 proteins that were enriched by greater than 10 counts in the ΔT2C CFS. [file elife-37172-supp1.docx]

Supplemental file 1. Mass spectrophotometry results comparing cell-free supernatant from the complement of the type II secretion mutant *A. veronii* strain Hm21 (WT, DT2C) compared to the isogenic type II secretion mutant (mut, DT2)

| Accession | Seq Length | kDa | Description | Difference WT vs mut |
| --- | --- | --- | --- | --- |
| F4DBG7_AERVB | 1007 | 109.316 | Metalloprotease stce OS=Aeromonas veronii (strain B565) GN=B565_0853 PE=4 SV=1 | 113 |
| F4DDC4_AERVB | **473** | **51.919** | **Chitin-binding protein, carbohydrate-binding module family 33 OS=Aeromonas veronii (strain B565) GN=B565_3557 PE=4 SV=1** | **112** |
| F4DC27_AERVB | 591 | 63.724 | Protease OS=Aeromonas veronii (strain B565) GN=B565_3414 PE=4 SV=1 | 105 |
| F4DBG8_AERVB | 638 | 69.583 | Chitinase, putative OS=Aeromonas veronii (strain B565) GN=B565_0854 PE=4 SV=1 | 72 |
| F4DAN4_AERVB | 830 | 91.892 | Putative trimethylamine-N-oxide reductase 1 OS=Aeromonas veronii (strain B565) GN=B565_3232 PE=4 SV=1 | 72 |
| F4DI47_AERVB | 1168 | 130.48 | Putative uncharacterized protein OS=Aeromonas veronii (strain B565) GN=B565_3977 PE=4 SV=1 | 60 |
| F4D853_AERVB | 919 | 103.622 | Collagenase family OS=Aeromonas veronii (strain B565) GN=B565_0474 PE=4 SV=1 | 53 |
| F4DE02_AERVB | 487 | 53.921 | Aeromonas virulence factor OS=Aeromonas veronii (strain B565) GN=B565_3626 PE=4 SV=1 | 45 |
| F4DHN3_AERVB | 624 | 66.694 | Serine protease Ahe2 OS=Aeromonas veronii (strain B565) GN=B565_1490 PE=3 SV=1 | 44 |
| F4DBG6_AERVB | 870 | 93.066 | Chitinase A OS=Aeromonas veronii (strain B565) GN=B565_0852 PE=3 SV=1 | 28 |
| F4DDA4_AERVB | 1354 | 145.483 | Pullulanase OS=Aeromonas veronii (strain B565) GN=B565_3537 PE=4 SV=1 | 24 |
| F4DAK7_AERVB | 574 | 61.732 | UshA protein OS=Aeromonas veronii (strain B565) GN=B565_3205 PE=3 SV=1 | 20 |
| F4DFH9_AERVB | **313** | **33.386** | **Putative uncharacterized protein OS=Aeromonas veronii (strain B565) GN=B565_0087 PE=4 SV=1** | **18** |
| F4DBH1_AERVB | 1009 | 107.821 | Chitinase OS=Aeromonas veronii (strain B565) GN=B565_0857 PE=4 SV=1 | 18 |
| F4DI46_AERVB | 729 | 79.011 | Glycoside hydrolase family 18 OS=Aeromonas veronii (strain B565) GN=B565_3976 PE=4 SV=1 | 13 |
| F4DFU7_AERVB | 641 | 70.456 | Twin-arginine translocation pathway signal OS=Aeromonas veronii (strain B565) GN=B565_1361 PE=4 SV=1 | 12 |
| F4DBH0_AERVB | 729 | 78.444 | Predicted extracellular nuclease OS=Aeromonas veronii (strain B565) GN=B565_0856 PE=4 SV=1 | 11 |
| F4DBG4_AERVB | 864 | 92.107 | Chitinase 92 OS=Aeromonas veronii (strain B565) GN=B565_0850 PE=3 SV=1 | 10 |
| … | … | … | … | … |
| F4DB22_AERVB | **328** | **34.875** | **Putative uncharacterized protein OS=Aeromonas veronii (strain B565) GN=B565_2031 PE=4 SV=1** | **6** |

Bold text indicates sizes of bands observed in the ammonium sulfate fractions with neutrophil reducing capacity.
