# Supplementary material for: A bacterial immunomodulatory protein with lipocalin-like domains facilitates host–bacteria mutualism in larval zebrafish
Source: eLife. 2018 Nov 6;7:e37172. doi: 10.7554/eLife.37172 (PMC6219842; doi:10.7554/eLife.37172)
Supplement: Supplemental file 2. — Structural homology searches performed against the N and C terminal domains of AimA separately revealed that both domains have similarity to proteins in the calycin superfamily, primarily avidins and lipocalins. [file elife-37172-fig2.docx]

Supplemental file 2. Structural homology hits to each AimA domain

| **AimA domain** | **Protein** | **Organism** | **PDB ID** | **Q score** | **RMSD** |
| --- | --- | --- | --- | --- | --- |
| Amino terminal  (19­ total hits) | Engineered Streptavidin | *Streptomyces avidinii* | 1kl3 | 0.17 | 2.69 |
|  | Avidin | *Gallus gallus* | 1lel | 0.16 | 3.56 |
|  | Zebavidin | *Danio rerio* | 4bj8 | 0.15 | 3.15 |
| Carboxy terminal  (220 total hits) | Avidin | *Gallus gallus* | 1lel | 0.30 | 2.58 |
|  | Zebavidin | *Danio rerio* | 4bj8 | 0.32 | 2.58 |
|  | Lipocalin lipoprotein | *Streptococcus pneumonia* | 5cyb | 0.29 | 2.28 |
|  | Lipoprotein | *Treponema pallidum* | 4u3q | 0.26 | 2.09 |
|  | Streptavidin  Retinol binding protein 4 | *Streptomyces avidinii*  *Homo sapiens* | 2izf  4o9s | 0.26  0.18 | 2.77  2.89 |
|  | Lipocalin-2 (LCN2, NGAL) | *Homo sapians* | 3i0a | 0.16 | 3.07 |
|  | Engineered Streptavidin | *Streptomyces avidinii* | 1kl3 | 0.18 | 2.69 |
|  | **Protein families** |  |  | **Q score range** | **RMSD range** |
|  | Avidins |  |  | 0.32-0.17 | 2.58-2.87 |
|  | Bacterial lipocalins | | | 0.29-0.21 | 2.28-2.85 |
|  | Non bacterial lipocalins |  |  | 0.26-0.16 | 2.09-3.07 |
